# The Role of Rho GTPases in Motility and Invasion of Glioblastoma Cells

**DOI:** 10.1155/2020/9274016

**Published:** 2020-01-31

**Authors:** Houssam Al-Koussa, Oula El Atat, Leila Jaafar, Hagop Tashjian, Mirvat El-Sibai

**Affiliations:** Department of Natural Sciences, School of Arts and Sciences, Lebanese American University, Beirut-, Lebanon

## Abstract

Astrocytomas are primary malignant brain tumors that originate from astrocytes. Grade IV astrocytoma or glioblastoma is a highly invasive tumor that occur within the brain parenchyma. The Rho family of small GTPases, which includes Rac1, Cdc42, and RhoA, is an important family whose members are key regulators of the invasion and migration of glioblastoma cells. In this review, we describe the role played by the Rho family of GTPases in the regulation of the invasion and migration of glioblastoma cells. Specifically, we focus on the role played by RhoA, Rac1, RhoG, and Cdc42 in cell migration through rearrangement of actin cytoskeleton, cell adhesion, and invasion. Finally, we highlight the importance of potentially targeting Rho GTPases in the treatment of glioblastoma.

## 1. Introduction

Primary brain tumors are a mixture of benign and malignant tumors originating from the brain parenchyma and its surrounding microenvironment [1]. Malignant brain and CNS tumors are the 11^th^ most common type of cancer and the 3^rd^ most common cause of cancer mortality [1, 2]. Annually, primary brain tumors occur at a frequency of around 7 per 100,000 individuals [2]. Approximately 70% of all primary brain tumors are classified as gliomas, which originate in glial cells. One group of gliomas, called astrocytoma, arises from astrocytes, which are a subtype of glial cells [2]. The WHO classifies astrocytoma into four grades (I-IV) according to their degree of malignancy, structural features, and genetic alterations. Grade IV astrocytoma, called glioblastoma multiforme (GBM), is the most common aggressive type of astrocytoma as well as the most lethal primary brain tumors. GBM has more pronounced malignant features, including uncontrolled cellular proliferation, increased angiogenesis, and resistance to apoptosis [1–3]. Therefore, patients diagnosed with GBM have a very poor prognosis with an average survival of 12-15 months post diagnosis [4, 5].

The invasive nature of GBM illustrates the molecular phenotype of cancer cells in general, which includes the constitutive activation of proinvasive pathways. The focus of this article is to discuss the role of Rho GTPase family proteins along with their upstream regulators and downstream effectors, which regulate the invasive and aggressive behavior of GBM through actin cytoskeleton rearrangement, cell adhesion, and invasion. Moreover, the potential ways through which Rho GTPases and associated proteins can be targeted for improved therapy will be further discussed.

## 2. Rho GTPases

Rho GTPases are involved in the regulation of cell growth, differentiation, cytoskeleton rearrangement, apoptosis, and cell motility [6]. The members of the Rho family of GTPases are small GTP-binding proteins with molecular sizes between 20 and 40 KDa [7]. These proteins act as molecular switches and exist in either an active GTP-binding or an inactive GDP-binding state [7]. They are activated by the guanine nucleotide exchange factors (GEFs), which, in turn, are activated downstream from PI3K in many cell types [8, 9]. Conversely, GTPase-activating proteins (GAPs) and guanine dissociation inhibitors (GDIs) lead to the inactivation of Rho GTPases [8]. Several members of the Rho family of GTPases have been in the context of cancer angiogenesis, migration, and invasion, most notably RhoA, RhoB, RhoC, RhoG, Rac1, and Cdc42 [10–14]. It has been established that RhoA plays a role in the formation of focal adhesion complexes and actin stress fibers while RhoG regulates cell migration through Rac1, which triggers the formation of lamellipodia and the appearance of membrane ruffles in many cell lines. Cdc42 is mainly involved in cell polarity and in the formation of filopodia [15, 16].

Briefly, Cdc42 relays environmental cues to effector proteins, setting the orientation of the cell. Rac and Rho are spatially regulated in opposite ways, which reflects their role in cell motility; at the front of the cell, Rac stimulates the formation of the leading edge while Rho governs the appearance and organization of contractile structures at the rear of the cell [13].

## 3. Altered Rho-GTPase Signaling in Glioblastoma

### 3.1. RhoA/RhoB/RhoC

RhoA, RhoB, and RhoC are Rho GTPase homologues that show an 88% similarity in the amino acid sequence. Each isoform has a distinct effect on the cell's structural and migration properties by activating different downstream effector proteins including enzymes and adaptor proteins [17]. Although they show a high level of structural similarity, these proteins have a very different intracellular distribution. RhoA and RhoC are found in the cytoplasm while RhoB is restricted to the endosome and plasma membrane. This distinct distribution allows them to interact with different target proteins [18, 19].

RhoA is a key regulator of cancer cell proliferation, progression, and metastasis. Several studies have shown that its activation leads to the formation of actin stress fibers and focal adhesions through RhoA-Rho-associated protein kinase (ROCK) signaling pathway [20, 21]. RhoB acts as a tumor suppressor by inhibiting tumor growth and inducing apoptosis in several types of cancer cells [22, 23]. Moreover, it has an effect on cancer cell adhesion and migration through the regulation of integrins [24]. Cancer cell metastasis is also regulated by RhoC expression, whereby knocking down RhoC has been reported to reduce cancer cell migration [25, 26].

RhoA is a well-established Rho GTPase that plays an important role in cell motility and invasion of glioblastoma and other tumor types. RhoA is activated upon binding of glioblastoma cells to the extracellular matrix (ECM) [27]. Following the interaction of integrins with the ECM, RhoA recruits actin, leading to the formation of nascent adhesions. These then becomes focal complexes, which are more stable and necessary adhesions that connect the lamellipodium to the lamella. This is referred to as early-stage adhesion [28–30]. At this point, Rho A activity is downregulated while that of Rac1 is upregulated. The focal complexes can either only serve for migration or mature to become focal adhesions, which are larger and stronger anchors to the ECM; this is known as late adhesion. These changes strengthen cell adhesion and hinder cell mobility [31]. In this case, Rho A activity is elevated and that of Rac1 is suppressed. Therefore, the switch between RhoA and Rac1 activation dictates whether the cell can separate and migrate or fixate and adhere to the ECM [32, 33]. In a study done on glioma cells, cell motility decreased following the overactivation of RhoA using oligodendrocyte lineage transcription factor 2 (OLIG2) [34]. Moreover, the expression of both RhoA and RhoB was tested in different types of brain cancer cells, and results showed that as the malignancy of the brain tumor increased, the levels of RhoA and RhoB expressions decreased [35].

RhoA induces the formation of focal adhesion (FA) by activating a serine/threonine kinase Rho effector p160ROCK. ROCK phosphorylates myosine light chain (MLC) by inhibiting the MLC phosphatase. The coordination between ROCK and the mammalian homolog of diaphanous formins (mDia), a RhoA effector, stimulates the formation of stress fibers and focal adhesion, enhancing cellular adhesion [36–39]. A study showed that the inhibition of ROCK in GBM cells leads to an increase in cellular motility. This is due to the appearance of membrane ruffling and the collapse of actin fiber [40].

RhoA activation leads to integrin clustering which promotes the activation of focal adhesion kinase (FAK) through phosphorylation at tyrosine 397. FAK signaling cascade regulates cell migration by controlling the focal complex's assembly and disassembly at the leading edge and the focal adhesions' (FA) disassembly at the trailing edge of the cell [41–43]. Focal adhesion complex and integrin bind to talin and paxillin in order to recruit FAK, which phosphorylates alpha-actin, an actin-binding protein, providing a force capable of moving the cell [44]. In a study done on glioblastoma cells, cerivastatin was used to inactivate FAK by disrupting the cytoskeleton, leading to the inhibition of migration [45].

For the cancer cell to remodel the ECM and facilitate invasion, metastasis, and survival, it needs to utilize matrix metalloproteinases (MMPs), a family of extracellular zinc-dependent proteinases [46, 47]. RhoA regulates MMP expression to activate cellular invasion [48]. A cell surface adhesion receptor, CD44, activates RhoA, inducing cellular invasion through the regulation of MMP-2 expression [48]. A study done on glioma cells provides further evidence that transmembrane metalloproteinase (MT1-MMP) is regulated by RhoA through the ROCK signaling pathway [49].

Moreover, RhoA is required in Rac1-induced lamellipodium formation at the leading edge of the invading cell [50]. The invasiveness of glioblastoma has been correlated with MMP expression. It has been shown that the expression levels of MMP-2 and MMP-9 are much higher in glioblastoma than less aggressive types of brain tumor [51, 52]. A study demonstrated that when MMPs were inhibited, glioma invasion was reduced [53]. When GBM was exposed to hypoxic conditions and bevacizumab, a VEGF sequestering drug that prevents angiogenesis, GBM, was able to utilize MMPs and invade the surrounding brain parenchyma tissues [54]. A recent study carried out on human GBM cells, using a new antimetastatic drug called ascochlorin (ASC), showed downregulation of MMP-2 activity and FAK signaling pathway, resulting in decreased cell migration and invasion [55].

RhoB plays a role in delivering receptors and signaling proteins to the plasma membrane through the process of endosomal trafficking [56]. RhoB depletion reduced cell spreading due to RhoB's role in cell surface integrin *β*1 trafficking. A study showed that downregulated levels of RhoB reduced surface *β*1 integrin levels [57]. This finding corroborates RhoB's role in integrin trafficking, highlighting the importance of RhoB in cell migration. Another study showed an overactivation of protein kinase C in glioblastoma, which caused the repression of RhoB, enhancing cell motility and invasion [58]. Furthermore, a different study done on glioblastoma cells showed that knocking down RhoB induced cell cycle arrest and activated apoptosis by activating p53 and p21 expressions [59].

Even though RhoA and RhoC have high sequence similarity, they regulate cellular migration differently. RhoC has been shown to induce cell migration and invasion by using a distinct signaling pathway from RhoA. A study done on prostate cancer showed that knocking down FMNL3, a protein found downstream of the RhoC signaling pathway, leads to the broadening of lamellipodia, depolarization of morphology, and inhibition of cellular invasion. Moreover, FMNL3 selectively binds to activated RhoC and not RhoA. This study also showed that RhoC regulates lamellipodium broadening by restricting Rac1 activation along the cellular membrane [60]. A different study was done on GBM cells infected with human cytomegalovirus (HCMV) in order to compare the role of the Rho GTPase isotypes RhoA, RhoB, and RhoC. This study showed that knocking down RhoA or RhoC will induce RhoB expression. Then, the study compared the effect of knocking down all three proteins separately and monitored their effect on cell migration. Results demonstrated that cells with RhoC knockdown had the fastest migration rate while those with RhoA knockdown had the slowest rate [61].

### 3.2. Rac1 and RhoG

The genetic profile of glioblastoma cells shows that most of them have more than one copy of chromosome 7, on which the Rac1 gene is located [62]. In the SNB19 glioma cell line, knocking down Rac1 leads to a drastic decrease in glioma cell migration and invasion [63]. Interestingly, knockdown of Rac3, a less commonly studied Rac protein, leads to the inhibition of invasion but not migration [63]. This observed functional divergence, despite their high sequence homology, can be due to their different subcellular localization.

The increased activity of Rac is the result of multiple factors including the deregulation of upstream regulators. Constitutive signaling through epithelial growth factor receptor (EGFR) and other receptor tyrosine kinases such as PDGFR*α*, caused by mutations and amplifications, activates Rac1, which in turn counters anti-EGFR treatment [62, 64]. This is most evident when targeting EGFR alone in GBM. Its therapeutic impact is limited due to multiple escape mechanisms, some of which include the presence of downstream signaling nodes [65]. Rac1 therefore is part of multiple signaling axes that start at receptor tyrosine kinases and that dictate cell migration and invasion. One such axis is the EGFR–DOCK180/ELMO1–RAC1–MLK3–JNK signaling axis which was found to be a driving factor of glioblastoma invasion [66]. DOCK180 is a Rac1 guanine nucleotide exchange factor (GEF) and is also downstream of other RTKs such as MET, forming a complex with several other proteins and driving Rac1-dependent glioblastoma cell movement [67]. Rho GEFs are discussed later in detail as both regulators of migration and invasion and as targets in therapy.

Another protein upstream of Rac1 is the TWEAK receptor Fn14, TWEAK being a TNF-like weak inducer of apoptosis. It was shown that TWEAK can play the role of a chemotactic signal by binding Fn14 and inducing cell movement in a Rac1-dependent manner [68]. TWEAK is also expressed by glial cells in the central nervous system, implying the presence of TWEAK signaled Rac1-dependent glioblastoma invasion [69]. This is further demonstrated by the fact that inhibition of the TWEAK/Fn14 signaling pathway in glioblastoma cells abolishes this chemotactic migration and invasion, as well as sensitizes cells to chemotherapy [70].

The other members of the Rac subfamily of Rho GTPases are not as well characterized in the context of glioblastoma and other brain tumors but can potentially be playing an important, yet still undeciphered, role in controlling migration and invasion of GBM cells. For example, in addition to Rac1, Rac2 and Rac3 knockdown in glioblastoma stem cells showed a dramatic decrease in their invasive and migrative capabilities, confirming that Rac proteins in general do play a crucial and necessary role in the invasion of GBM cells [71].

RhoG, a Rac superfamily member, regulates cell polarity, migration, and invasion. It has been reported that RhoG, activated by EGF and hepatocyte growth factor (HGF), contributes to the formation of lamellipodia and invadopodia and promotes glioblastoma cell migration and invasion [72]. Knocking down RhoG partially inhibited lamellipodium and invadopodium formation and abolished cell invasion. In fact, RhoG enhances cell migration and invasion through Rac1-dependent and Rac1-independent pathways [72]. Moreover, the TWEAK-Fn14 signaling discussed previously stimulates RhoG-dependent Rac1 activation and subsequently rebuts lamellipodium formation and enhances the invasive behavior of glioblastoma [73]. Recently, research in our lab demonstrated that RhoG positively regulates cell adhesion, migration, and invasion through the activation of Rac in glioblastoma cells [74].

### 3.3. Cdc42

Findings regarding the role of Rac1 in glioma cell migration and invasion parallel those of Cdc42, for both, promote these two processes, bind to similar domains, are activated by common GEFs, and have the same intracellular location (at the front edge of the cell). High expression of Cdc42 in GBM is correlated with lower survival rates, since it was found to promote the highly invasive characteristics of GBM in vivo [75].

A close inspection of its subcellular localization reveals several structures and proteins with which this Rho GTPase possibly interacts. One such protein is IQGAP1, a scaffold protein which binds both Rac1 and Cdc42 and helps reorganize actin cytoskeletal structures [76]. Knockdown of IQGAP1 inhibits both Rac1- and Cdc42-mediated migration and invasion of glioma cells and leads to the suppression of several other components of the invasion process such as MMPs [77]. It is therefore not surprising that in addition to Cdc42, high levels of proteins that interact with Cdc42 like IQGAP1 are correlated with poor survival of patients with glioma [78].

Cdc42 is also believed to be a mediator of the X-ray promoted invasion of glioblastoma, a complication that occurs following radiotherapy. Cathepsin L was found to be a strong promoter of X-ray-induced glioma cell invasion by regulating Cdc42-mediated cytoskeletal remodeling, a process which was abolished once Cathepsin L was knocked down and Cdc42 levels decreased [79]. Radiation-induced invasion is also molecularly different than other forms of invasion, a fact which can help stratify and target glioblastoma according to circumstances [80].

Another important aspect of the implication of Cdc42 in the malignancy of GBM is the interaction between GBM cells and other cells in the tumor environment, specifically pericytes. In this context, Cdc42 was found to promote the development of previously unnamed structures now called flectopodia (due to their significant differences from “classical” invadopodia) which establish communication with pericytes, turning the tumor-suppressive nature of these regulatory cells into tumor promoting and clearing the way for cancer cell invasion [81].

The levels of Rac1 and Cdc42 are not the same throughout the cell. According to a biosensor assay done on glioblastoma cells, low Rac1 and low Cdc42 are found on the trailing end while high Rac1 and high Cdc42 levels are found on the leading edge of the cells [82]. RhoA, Rac1, and Cdc42 levels are not the only factors affecting invasiveness and migration; it is the crosstalk between different Rho GTPases that establishes the harmonious signaling of cell movement. Upstream regulators of this balance are necessary for GBM migration and invasion, and their absence abolishes this balance. As such, following the knockdown of *β*8 integrin, a Rho-GDI sequestering membrane protein, and despite the increase in overall Cdc42 levels, migration and invasion are diminished [83]. Hence, there exist signaling axes carefully regulating the optimal activity of Rho GTPases.

## 4. Upstream Regulators

### 4.1. Rho GEFs

Rho GEFs promote the GDP to GTP switch which activates Rho GTPases. In agreement of our understanding of the role of Rho GTPases is the fact that Rho GEFs are involved in glioma cell migration and invasion. This also provides a theoretical basis for targeting the Rho/Rho GEF interaction as a way to inhibit GBM cell migration.

The expression of the three GEFs Ect2, Trio, and Vav3 is elevated in the glioblastoma as compared to lower-grade glioma, and the depletion of any of the three is sufficient to reduce migration and invasion of glioblastoma [84]. The Rho GEF Ect2 mediates Cdc42 activation and, subsequently, the activation of Rac1. Trio mediates Rac1 activation following TWEAK stimulation in glioblastoma cells [85]. Furthermore, RhoG-specific exchange factor SGEF mediates RhoG activation, which in turn activates Rac1 in glioblastoma cells [73].

Another Rho GEF is SWAP-70 which is overexpressed in human high-grade glioma tissues and high-grade glioma patients [86]. Following EGF stimulation, SWAP-70 was detected at the leading edge of migrated glioma cells, accompanied by an increase in Rac1 activity [87]. On the other hand, dedicator of cytokinesis 1 (DOCK1), also known as DOCK180, is a Rac GEF which associates with cofactor engulfment and cell motility 1 (ELMO1) overexpressed in glioma cells and activates Rac1 [88]. The stimulation of PDGFR*α* by PDGF growth factor leads to the phosphorylation of DOCK1 which, in turn, activates Rac1 and subsequently enhances glioma cell migration and invasion [89]. Of note is that DOCK1 also plays a central role in the aforementioned EGF-promoted EGFR–DOCK180/ELMO1–RAC1–MLK3–JNK signaling axis [66]. Additionally, DOCK 7 is reported to play a critical role in the invasive behavior of the glioblastoma and mediates Rac1 activation following HGF stimulation [90]. Another Rac GEF that promotes Rac1-mediated invasion in glioblastoma cells is PREX1, which is overexpressed in GBM and responds to the presence of the upregulated Pi3K pathway [91].

Recently, novel Rho GEFs were discovered and they have been implicated in the aggressive phenotype of GBM. Overexpression of PDZ-Rho GEF in TROY-expressing glioblastoma cells mediates RhoA and RhoC activation downstream of the TROY receptor [92]. Moreover, Pleckstrin homology and Rho GEF domain containing G5 (PLEKHG5) was characterized as a prognostic biomarker for glioma patients. It was found to be overexpressed in the glioblastoma when compared to low-grade glioma cells. Depletion of PLEKHG5 decreases the expression of matrix metalloproteinases MMP-2 and MMP-9 and the expression of mDia, which suggests its importance in mediating cell migration and invasion by regulating actin assembly and proteolytic enzyme secretion [93].

### 4.2. PTEN and Rho GAPs

The loss or dysregulation of certain tumor suppressors is associated with the Rho-mediated aggressive behavior. We will briefly reflect on a couple of them. One of these proteins is phosphatase and tensin homolog (PTEN), which suppresses phosphatidylinositol-3,4,5-trisphosphate (PIP3) level cells. Loss or mutations of PTEN correlate with poor patient outcome in GBM [94, 95], and depending on the study, PTEN is mutated in 5-40% of glioblastoma patients, and loss of heterozygosity can be observed in up to 90% of cases [96–100]. This, in addition to increased PI3K activity in many GBMs, leads to high levels of PIP3. Multiple Rho GEFs, including DOCK1 and PREX1, are either stimulated or require PIP3 binding for their activation [101, 102]. These conditions in PTEN-deficient cells therefore favor the activation of Rho GEFs and by extension the much discussed signaling that occurs in the glioblastoma.

Rho GAPs are Rho-negative regulators that inhibit Rho activity and abolish glioma cell migration and invasion. Neurofibromin 1 (NF1) is a GAP specific for Ras GTP, which occupies a key signaling node of the Ras/Raf/MAPK oncogenic pathway as well as crosstalks with the Pi3K pathway upstream of Rho proteins [103]. NF1 also directly inhibits the Rho/ROCK/LIMK2/cofilin pathway by binding LIMK2 and preventing ROCK activation of the pathway [104]. LIMK is upregulated in the glioblastoma, and LIMK2 knockdown tumor xenografts were found to be much less invasive than the control [105, 106]. It is interesting to note these facts because NF1 gene is inactivated by one of many mechanisms in about 13% of glioblastoma, and NF1 loss is directly correlated with GBM aggressiveness [107, 108]. Loss of NF1 therefore means upregulation of Rho and Rho-mediated invasive signals. ARHGAP21a is a Rho GAP that reduces the aggressive phenotype of GBM by inactivating Cdc42 and FAK → p130^CAS^ and decreasing MMP-2 [109]. It has been reported that the expression of ARHGAP15 is reduced in high-grade glioblastoma. Once overexpressed, it inhibits glioma cell migration and invasion through the inactivation of Rac1. Sun et al. demonstrated that the activity of FocP3, an EMT regulator, is partially mediated by ARHGAP 15 through Rac1 inactivation [110]. Furthermore, P190RhoGAP was found to inactivate RhoA following SEMA3F-ABL2/ARG tyrosine kinase interaction (signaling) [111]. Finally, the Rac-specific GAP FilGAP mediates Rho/ROCK-dependent amoeboid migration and might contribute to impaired glioma cell migration. Following the silencing of FilGAP, the level of expression of Rac1 slightly increases, and the number of mesenchymal-like cells increases significantly. Therefore, FilGAP/Rac1 axis may be considered a regulator for GBM invasive behavior [112].

## 5. Targeted Therapy

After studying the role of Rho GTPases in glioblastoma, therapy focuses on the search for druggable proteins and strategies that could target Rho GTPase interaction with Rho GEFs, their downstream effectors, or even their degradation. Most of the following drugs have antiproliferative effects, but in this review the major focus is on cell migration.

NSC23766 is a Rac1 inhibitor that synergistically inhibits glioma cell migration and invasion when used with anti-EGFR drugs [113]. This Rac1-GEF interaction inhibitor is selective towards Rac1 and does not inhibit the interaction of the closely related Cdc42 and RhoA with GEFs [114]. Other Rac1-GEF interaction inhibitors exist and are currently in development, such as ZINC69391 and its potent anti-invasive analog 1A-116 [114], and Rac1 inhibitors continue to show synergistic effects when combined with anti-EGFR drugs [115].

Another possible way through which new drugs can act is through the downstream effectors of Rho GTPases. In this regard, resveratrol was found to suppress glioblastoma cell migration and invasion by increased activation of the RhoA/ROCK pathway [116]. Resveratrol is also known to increase cancer cell sensitivity to chemotherapy such as temozolomide, the standard chemotherapeutic agent used in glioblastoma treatment [117]. In the RhoA/mDia pathway, it was a mDia agonist that inhibited all movement in U251 cells. These results support the development of agonistic strategies to inhibit cell migration [118].

Another way to modulate the levels of Rho GTPase is targeting them for ubiquitination. In this context, luteolin was found to facilitate the proteasomal degradation of Cdc42, leading to decreased glioblastoma migration and invasion in vitro [119]. This illustrates the diverse ways in which Rho GTPases can be targeted in cancer therapy. Other drugs can target the spatial regulation of Rho proteins by interfering with posttranslational modifications necessary for Rho GTPase activity [120].

Although major limitations persist from the perspective of drug delivery, we hypothesize that future development and usage of Rho GTPase signaling inhibitors in combination with standard therapy has the potential to improve outcome for patients with glioblastoma.

## 6. Conclusion

The aggressive nature of glioblastoma impairs most of the attempts aimed at curbing disease progression in patients. Research has enabled the understanding of the function of Rho GTPases in cancer and the interaction of cancers with their surroundings. Given the involvement of Rho GTPases in GBM migration, invasion, and aggressiveness, Rho GTPases, as well as their upstream regulators and downstream effectors are emerging as strong candidates for cancer treatment.
